# Anatomy of the Human Subthalamic Nucleus: A Combined Morphometric Study

**DOI:** 10.1155/2013/319710

**Published:** 2013-12-15

**Authors:** Ioannis Mavridis, Efstathios Boviatsis, Sophia Anagnostopoulou

**Affiliations:** ^1^Department of Anatomy, University of Athens, School of Medicine, Mikras Assias street 75, Goudi, 11527 Athens, Greece; ^2^2nd Department of Neurosurgery, Attikon Hospital, University of Athens, School of Medicine, Rimini Street 1, Haidari, 12462 Athens, Greece

## Abstract

*Purpose*. Our purpose was to provide a combined clinically oriented study focused on the detailed anatomy of the human STN, with great respect to its targeting. *Methods*. For our imaging study, we used cerebral magnetic resonance images (MRIs) from 26 neurosurgical patients and for our anatomic study 32 cerebral hemispheres from 18 normal brains from cadaver donors. We measured and analyzed the STN dimensions (based on its stereotactic coordinates). *Results*. At stereotactic level *Z* = −4, the STN length was 7.7 mm on MRIs and 8.1 mm in anatomic specimens. Its width was 6 mm on MRIs and 6.3 mm in anatomic specimens. The STN was averagely visible in 3.2 transverse MRI slices and its maximum dimension was 8.5 mm. The intercommissural distance was 26.3 mm on MRIs and 27.3 mm in anatomic specimens. We found statistically significant difference of the STN width and length between individuals <60 and ≥60 years old. *Conclusion*. The identification of the STN limits was easier in anatomic specimens than on MRIs and easier on T2 compared to T1-weighted MRIs sections. STN dimensions appear slightly smaller on MRIs. Younger people have wider and longer STN.

## 1. Introduction

The human subthalamic nucleus (STN) is a massive biconvex lens-shaped nucleus located under the thalamus. Among the basal ganglia nuclei, the STN has a major function in the motor cortico-basal ganglia-thalamocortical circuit and is a target site for neurosurgical treatment such as parkinsonian patients with long-term motor fluctuations and dyskinesia [1].

The motor functions of the STN were established in humans from clinical observations of contralateral hemiballism induced by STN ischemia. Its motor role was confirmed by metabolic, electrophysiological, and behavioral studies performed in healthy animals and animals' models of Parkinson's disease (PD) [1]. Experimental studies in animals and clinical observations of parkinsonian patients showed that the STN had also associative and limbic functions. After STN stimulation, some patients became apathetic or depressed or had impaired recognition of facial emotion [1].

Although the role of the STN in the motor cortico-basal ganglia-thalamocortical loop is well known, its implication on limbic functions remains to be described by a new scheme of the limbic loop including the STN. After chronic STN stimulation in patients with PD, many studies showed executive impairments, apathy, depression, hypomania, and impairment of recognition of negative facial emotions. The medial tip of the STN represents its limbic part. This part receives inputs from the anterior cingulate cortex, the medial prefrontal cortex, the limbic part of the striatum (nucleus accumbens), the ventral tegmental area, and the limbic ventral pallidum. The medial tip of the STN projects to the limbic part of the substantia nigra and the ventral tegmental area [1].

The purpose of our clinically oriented anatomical-radiologic study was to provide anatomical and imaging data of the human STN, primarily useful to neurosurgeons. Therefore our objectives were to reveal the STN location, making surgically interesting brain sections and to measure its dimensions. We also measured STN dimensions at the same levels on magnetic resonance images (MRIs).

## 2. Methods

### 2.1. Imaging Study

For our imaging study we used cerebral MRIs (1.5 Tesla, T1-weighted coronal, transverse and sagittal 2 mm sections, as well as T2-weighted transverse 2 mm sections) from 26 neurosurgical patients (16 males, 23–70 years old and 10 females, 38–71 years old), from our second author's personal archive. These MRIs were carried out during the period 2005–2009 and these patients had no obvious brain pathology (e.g., space-occupying lesions) able to affect the STN location and size.

**Figure 1:**
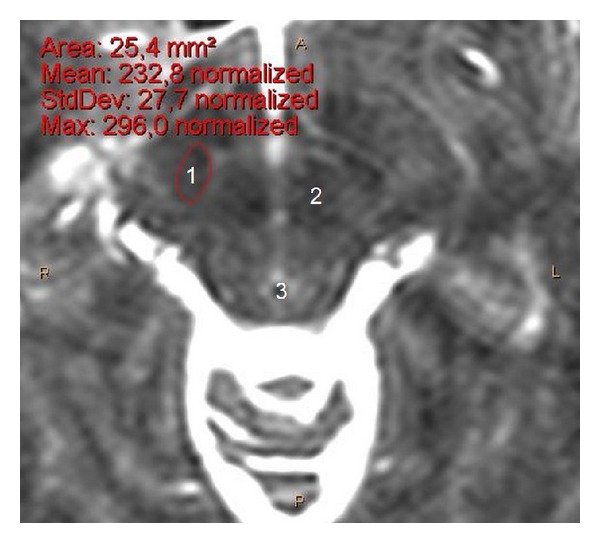
Transverse cerebral MRI section (T2-weighted, 1.5 Tesla) at level *Z* = −4 (zoom on mesencephalon), from a 37-year-old male, showing the STN location and surface area. 1: STN, 2: red nucleus, 3: aqueduct (of Sylvius), A: anterior, P: posterior and, R: right, L left.

**Figure 2:**
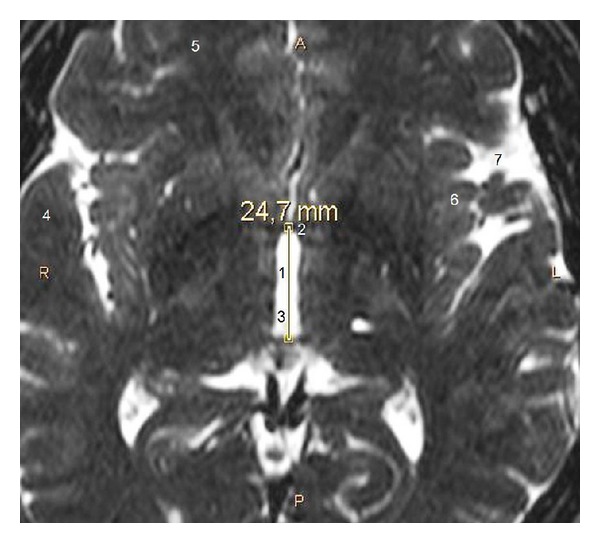
Transverse cerebral MRI section (T2-weighted, 1.5 Tesla) at level *Z* = 0, from a 51-year-old male, showing measurement of the AC-PC length. 1: AC-PC length, 2: AC, 3: third ventricle, 4: temporal lobe, 5: frontal lobe, 6: insula, 7: Sylvian fissure, A: anterior, P: posterior and, R: right, L left.

We measured the *X*, *X*′, *Y*, *Y*′, *Z*, *Z*′ stereotactic coordinates of the STN at the following levels: transverse level 4 mm ventrally to the anterior-posterior commissure (AC-PC) plane (*Z* = −4) (Figure 1), coronal level 2 mm posterior (*Y* = −2) to the midcommissural point (MCP), and sagittal level 12 mm lateral to the midline (*X* = 12). In both of our studies, the MCP (a midline point, equally distant from the AC and PC) defines the reference point with stereotactic coordinates (*X*, *Y*, *Z*) = (0,0, 0). The STN width was measured as *W* = *X*′ − *X*, the STN height as *H* = *Z* − *Z*′, and the STN length as *L* = *Y* − *Y*′. We also measured the maximum transverse dimension (diameter) *D* of the STN (regardless of level), as well as AC-PC length (Figure 2).

We followed the same methodology of measuring in all cases (using Philips DICOM Viewer) and all measurements (50 STNs) were made by the same author for more objective results. Moreover, we identified the specific transverse, coronal, and sagittal levels where each STN presented its maximum size. We statistically analyzed our results and made comparisons among side, gender, and age (<60 and ≥60 years old) using Student's *t*-test.

### 2.2. Anatomic Study

The material consisted of 26 cerebral hemispheres (17 left and 9 right) from 17 normal human brains which we have in our department (Department of Anatomy). They come from 16 males, 50–60 years old, and one female, 94 years old, cadaver donors for students' education. These brains have been fixed in formalin solution for a short time (in order to avoid, as much as possible, potential morphological changes).

**Figure 3:**
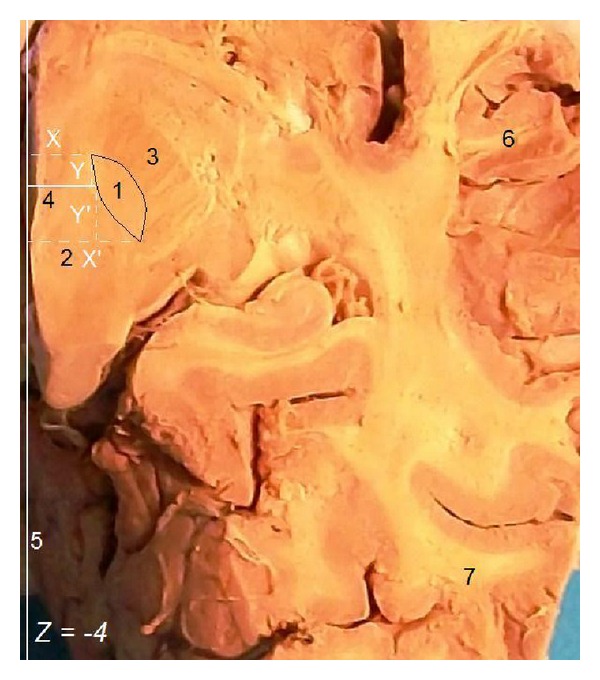
Formalin-fixated human brain from a middle-aged male, right cerebral hemisphere, transverse section at *Z* = −4 showing measurement of the STN coordinates. 1: STN, 2: red nucleus, 3: cerebral peduncle, 4: coronal level (perpendicular to the AC-PC plane) containing the MCP, 5: midline, 6: temporal lobe, 7: occipital lobe, *X*, *X*′, *Y*, *Y*′, *Z* stereotactic coordinates of the STN.

Methodologically, first we found the location of the AC, PC, and MCP at the internal hemispheric surface and we noticed, with a scalpel blade, the intercommissural (AC-PC) plane as well as the, perpendicular to the AC-PC plane, coronal plane containing the MCP. Then we made our transverse sections at the transverse level 4 mm ventrally to the AC-PC plane (*Z* = −4). Using the same reference point, the *X*, *X*′, *Y*, *Y*′ stereotactic coordinates of the STN were identified at *Z* = −4 (Figure 3) and the *W*, *L*, and *D* of the STN were calculated. Moreover, we measured AC-PC length and our anatomic results were also statistically analyzed.

## 3. Results

### 3.1. Imaging Study

#### 3.1.1. Transverse Sections

Table 1 shows values of the STN *W* (*n* = 45) and *L* (*n* = 46) at level *Z* = −4, as well as mean values (MVs) and their standard deviations (SDs). Information regarding *D*, age, gender, AC-PC length, and the number of transverse slices where the STN was visible is also presented.

MV ± SD of each dimension for right STNs was *W* = 6.08 ± 1.50 mm (*n* = 22), *L* = 7.81 ± 1.53 mm (*n* = 23), and *D* = 8.55 ± 1.52 mm (*n* = 24). The mean number of slices where the right STN was visible was found to be 3.16 ± 0.47. The minimum observed *W* was 4.0 mm and the maximum was 8.9 mm. The minimum observed *L* was 4.9 mm and the maximum was 11.2 mm. The minimum observed *D* was 5.6 mm and the maximum was 12.1 mm.

MV ± SD of each dimension for left STNs was *W* = 5.88 ± 1.44 mm (*n* = 23), and *L* = 7.66 ± 1.63 mm (*n* = 23), *D* = 8.49 ± 1.64 mm (*n* = 23). The mean number of slices where the left STN was visible was found to be exactly the same as for the right STNs. The minimum observed *W* was 4.0 mm and the maximum was 8.8 mm. The minimum observed *L* was 4.8 mm and the maximum was 9.4 mm. The minimum observed *D* was 4.8 mm and the maximum was 11.6 mm. No statistically significant difference of each MV was found between the two sides.

MV ± SD of each dimension for male STNs was *W* = 5.93 ± 1.39 mm (*n* = 29), *L* = 7.72 ± 1.63 mm (*n* = 30), and *D* = 8.48 ± 1.67 mm (*n* = 31). The mean number of slices where the male STN was visible was found to be 3.19 ± 0.54. AC-PC length was 26.16 ± 3.35 mm (*n* = 16). The minimum observed *W* was 4.0 mm and the maximum was 8.7 mm. The minimum observed *L* was 4.8 mm and the maximum was 11.0 mm. The minimum observed *D* was 4.8 mm and the maximum was 12.1 mm. The minimum AC-PC length was 17.6 mm and the maximum was 31.0 mm.

MV ± SD of each dimension for female STNs was *W* = 6.08 ± 1.63 mm (*n* = 16), *L* = 7.76 ± 1.49 mm (*n* = 16), and *D* = 8.61 ± 1.38 mm (*n* = 16). The mean number of slices where the female STN was visible was found to be 3.11 ± 0.32. AC-PC length was 26.55 ± 1.03 mm (*n* = 10). The minimum observed *W* was 4.0 mm and the maximum was 8.9 mm. The minimum observed *L* was 5.6 mm and the maximum was 11.2 mm. The minimum observed *D* was 6.5 mm and the maximum was 11.0 mm. The minimum AC-PC length was 25.0 mm and the maximum was 27.9 mm. No statistically significant difference of each MV was found between the two sexes.

MV ± SD of each dimension for individuals **<**60 years old was *W* = 6.49 ± 1.41 mm (*n* = 23), *L* = 8.18 ± 1.56 mm (*n* = 24), and *D* = 8.78 ± 1.54 mm (*n* = 26). The mean number of slices where the “young” STN was visible was found to be =3.07 ± 0.47. AC-PC length was 26.88 ± 1.35 mm (*n* = 15). The minimum observed *W* was 4.1 mm and the maximum was 8.9 mm. The minimum observed *L* was 4.8 mm and the maximum was 11.2 mm. The minimum observed *D* was 6.0 mm and the maximum was 12.1 mm. The minimum AC-PC length was 24.7 mm and the maximum was 28.5 mm.

For individuals ≥60 years old these values were *W* = 5.45 ± 1.34 mm (*n* = 22), *L* = 7.25 ± 1.45 mm (*n* = 22), and *D* = 8.20 ± 1.56 mm (*n* = 21). The mean number of slices where the “old” STN was visible was found to be 3.27 ± 0.46. AC-PC length was 25.53 ± 3.70 mm (*n* = 11). The minimum observed *W* was 4.0 mm and the maximum was 8.7 mm. The minimum observed *L* was 5.4 mm and the maximum was 11.0 mm. The minimum observed *D* was 4.8 mm and the maximum was 11.6 mm. The minimum AC-PC length was 17.6 mm and the maximum was 31.0 mm. We found statistically significant difference of the *W* MV (*t* = 2.55, *P* < 0.05, df = 43) between individuals <60  (6.49 ± 1.41) and ≥60 years old (5.45 ± 1.34). Statistically significant difference of the *L* MV (*t* = 2.11, *P* < 0.05, df = 44) was also found between individuals <60  (8.18 ± 1.56) and ≥60 years old (7.25 ± 1.45).

#### 3.1.2. Coronal Sections

MV ± SD of each STN dimension at level *Y* = −2 was: *W* = 8.13 ± 1.15 mm (*n* = 4), *H* = 4.98 ± 0.56 mm (*n* = 4), *D* = 8.22 ± 0.53 mm (*n* = 6). The mean number of slices where the STN was visible found to be 4.33 ± 0.52. The minimum observed *W* was 6.6 mm and the maximum was 9.2 mm. The minimum observed *H* was 4.2 mm and the maximum was 5.5 mm.

#### 3.1.3. Sagittal Sections

MV ± SD of each STN dimension at level *X* = 12 was *H* = 5.42 ± 1.38 mm (*n* = 6), *L* = 7.70 ± 0.96 mm (*n* = 6), and *D* = 6.79 ± 1.44 mm (*n* = 12). The mean number of slices where the STN was visible was found to be 4.75 ± 0.46. The minimum observed *H* was 3.8 mm and the maximum was 7.1 mm. The minimum observed *L* was 6.9 mm and the maximum was 9.5 mm. For the right STNs, we found *D* = 7.00 ± 1.55 mm (*n* = 6) and for the left *D* = 6.58 ± 1.43 mm (*n* = 6). No statistically significant difference of the *D* MV was found between the two sides.

### 3.2. Anatomic Study

Table 2 shows values of AC-PC length and STN dimensions *W*, *L*, and *D* at level *Z* = −4 as well as MVs and their SDs. MV ± SD of STN *W* was *W* = 6.29 ± 1.65 mm (*n* = 24), MV ± SD of STN *L* = 8.08 ± 1.50 mm (*n* = 24), MV ± SD of STN *D* = 9.23 ± 1.37 mm (*n* = 24) and MV ± SD of AC-PC length = 27.31 ± 2.59 mm (*n* = 26).

For the right STNs, we found *W* = 6.50 ± 1.20 mm (*n* = 8), *L* = 7.88 ± 1.55 mm (*n* = 8) and *D* = 9.00 ± 1.77 mm (*n* = 8). The minimum observed *W* was 5.0 mm and the maximum was 9.0 mm. The minimum observed *L* was 5.0 mm and the maximum was 10.0 mm. The minimum observed *D* was 6.0 mm and the maximum was 11.0 mm.

For left STNs we found *W* = 6.19 ± 1.87 mm (*n* = 16), *L* = 8.19 ± 1.52 mm (*n* = 16), and *D* = 9.34 ± 1.17 mm (*n* = 16). The minimum observed *W* was 3.0 mm and the maximum was 10.0 mm. The minimum observed *L* was 6.0 mm and the maximum was 11.0 mm. The minimum observed *D* was 7.0 mm and the maximum was 11.0 mm. No statistically significant difference of each MV was found between the two sides.

## 4. Discussion

### 4.1. Imaging Study

In both our imaging and anatomic studies, we chose the MCP as the reference point with stereotactic coordinates (*X*, *Y*, *Z*) = (0,0, 0), because it is the most often reported reference point used for deep brain stimulation (DBS) of the STN [2–17]. The three-dimensional (3D) levels of our combined study were chosen after careful review of the worldwide existing clinical experience of STN DBS. Hence these levels are, as explained below in detail, clinically important.

### 4.2. Transverse Sections

Coordinate *Z* = −4 ± 0,5 mm has been repeatedly reported as coordinate of the electrode target point for STN DBS [2–4, 7–9, 11, 12, 14–16]. Hence we chose the level *Z* = −4 for both our imaging and anatomic studies of the STN.

The statistically significant difference of the MV of the STN *W*, we found, between individuals <60  (6.49 ± 1.41) and ≥60 years old (5.45 ± 1.34), indicates that the STN is wider in younger people. The statistically significant difference of the MV of the STN *L* between individuals <60  (8.18 ± 1.56) and ≥60 years old (7.25 ± 1.45), indicates that the STN is longer in younger people. These data suggest that the STN suffers age-related shrinkage.

Shen et al. [18] investigated the 3D target location of STN in stereotactic space and constructed a digitalized atlas of STN to accomplish the visualization of the STN on stereotactic MRI, thus providing clinical guidance on the precise anatomical localization of STN. 120 healthy people volunteered to be scanned by 1.5 Tesla MRI scanning with 1 mm thick slice in the standard stereotactic space. One adult male was selected for 3D reconstruction of the STN. The left STN volume was found to be significantly larger than that of the right STN, and there was a significant negative correlation between volume and age (*P* < 0.05). The anteroposterior diameter of the STN was longer than the vertical and transverse diameters in 3D space [18]. Except for the volumetric difference among side, our results are in agreement with those of Shen et al.

According to our experience, the definition of the STN limits is easier on T2-weighted MRIs, due to the slightly more intense MR signal that this nucleus presents compared to T1-weighted sections.

### 4.3. Coronal Sections

Coordinate *Y* = −2 ± 0,5 mm has been repeatedly reported as coordinate of the electrode target point for STN DBS [3–6, 10, 13, 14, 16, 17]. Hence we chose the level *Y* = −2 for our imaging study of the STN. *Z* and *Z*′ coordinates were not measured at this level because of the difficulty to identify the AC-PC plane precisely in these sections. In coronal and sagittal sections, we avoided comparisons among side, gender, and age because of the relatively small (at least compared with our transverse sections) number of STNs studied.

### 4.4. Sagittal Sections

Coordinate *X* = 12 ± 0,5 mm has been repeatedly reported as coordinate of the electrode target point for STN DBS [2, 3, 7–17]. Hence we chose the level *X* = 12 for our imaging study of the STN.

### 4.5. Anatomic and Comparative Study

The definition of the STN limits found to be easier on gross anatomic specimens than on MRIs. The STN dimensions (*W*, *L*, *D*) we found in our anatomic specimens, as well as AC-PC length, are comparable with our radiologic results. We consider our anatomic results as quite reliable because of our ability of “touch on” measurements. Interestingly, all our anatomic values were slightly greater than the respective imaging values. The mean AC-PC length was exactly 1 mm longer in anatomic specimens than on MRIs (27.31 ± 2.59 versus 26.31 ± 2.67).

Daniluk et al. [19] used 62 axial T2-weighted preoperative (before STN-DBS) cerebral MRIs (1.5 Tesla) from 62 PD patients (37 males) to obtain MCP-derived coordinates of STN borders, STN center, and other brain landmarks. MR-derived measurements were compared to Schaltenbrand and Wahren Atlas. They evaluated 117 STNs and found its dimensions and coordinates of its center to be highly variable. We agree with them that it is possible to directly evaluate STNs at 1.5 Tesla with minimal image distortion, which reveals variation in STN position and dimensions [19].

According to their results [19], MV ± SD of STN *L* was 9.4 ± 1.27 mm, ranging from 5.2 to 13.2 mm, while Schaltenbrand and Wahren Atlas value was 10.1 mm. MV ± SD of STN *W* was 9.1 ± 1.13 mm, ranging from 6.4 to 12.2 mm, while Schaltenbrand and Wahren Atlas value was 10.1 mm. MV ± SD of AC-PC length was 26.3 ± 1.81 mm, ranging from 22.9 to 29.9 mm, while Schaltenbrand and Wahren Atlas value was 20.5 mm. Although their values, regarding STN dimensions, were greater than ours (*L* and *W*: 7.73 mm and 5.98, resp.), we have absolute agreement about AC-PC length.

STN *L* dimension for males was 9.7 ± 1.33 mm and for females 9.1 ± 1.11 mm (*P* < 0.05). STN *W* for males was 9.3 ± 1.08 mm and for females 9.0 ± 1.19 mm. AC-PC length for males was 26.9 ± 1.51 mm and for females 25.3 ± 1.80 mm (*P* < 0.001) [19]. In contrast to these results, we observed no significant difference between the two sexes.

STN *L* was significantly correlated with STN *W* (*r* = 0.35, *P* < 0.001) and with biputamen distance at MCP. AC-PC length was significantly correlated with brain *L* at MCP, brain *W* at MCP, biputamen distance at MCP, and third ventricle *W* at MCP. Furthermore, the mean MR-derived dimensions were smaller than dimensions shown in the atlas [19]. The latter observation (smaller MRI than gross anatomic dimensions) was confirmed by our comparative study.

Richter et al. [20] studied both the STN size and location based on MRIs, compared with those on the Talairach and Tournoux and Schaltenbrand and Wahren atlases. The STN position relative to the MCP was evaluated on 18 T2-weighted MRIs (35 evaluable STNs), 2 mm slices. These methods were validated using histological measurements in one case in which a postmortem examination was performed. The mean AC-PC length was 25.8 mm. The STN was smaller on MRIs compared with its size in atlases in the anteroposterior (mean 5.9 mm), mediolateral (3.7 mm), and dorsoventral (5 mm) dimensions [20]. Although our results confirm the latter observation, the MVs we found regarding STN *L* and *W* are significantly greater (7.73 mm and 5.98, resp.). However, we agree that STN *L* > STN  *W*.

Caire et al. [21] aimed to compare the STN position localized on MRIs with standard stereotactic diagrams. The STN was manually contoured on MRIs (22 PD patients); boundaries were simplified in a schematic polygonal form. Front and lateral stereotactic diagrams were constructed according to Talairach and Benabid. There was significant discordance between MRI-based polygons and AC-PC-based images. MRI showed the STN as more posterior, medial, and slightly inferior. The density maps showed discordance between the locations of MRI anatomy STN polygons and stereotactic AC-PC-based STN diagrams, regardless of whether the stereotactic reference space was Talairach or Benabid. Specifically, on the lateral view, MRI anatomy-based polygons were located more inferiorly and posteriorly, whereas on the front view they were located more inferiorly and medially [21].

Lange et al. [22] reported a morphometric statistical analysis of the STN, carried out on 14 hemispheres (12 normal human brains). The values determined in paraffin embedded sections were corrected for shrinkage which showed considerable interindividual variation. The mean fresh volume of the STN amounted to 144 mm^3^ for males and 134 mm^3^ for females (the difference of 7% not being statistically significant). The STN occupied 0.027% of the volume of the hemisphere [22].

According to both Talairach et al. [23] and Benabid et al. [24] the AC-PC length has a mean value of approximately 25 mm (24.7 ± 1.57 mm and 24.89 ± 1.54 mm, resp.).

### 4.6. Considerations

The strengths of our study contain the total number of STNs studied (75) and its combined character. To our knowledge, our work consists of the most extensive gross anatomic study of the human STN. As a weakness, we should mention that our anatomic data were obtained on formalin-fixed specimens and transferred to the in vivo situation (an expected issue in anatomic studies). Our particular care in reducing the formalin effect to the specimen was to minimize, as much as possible, the fixation time. However, as already mentioned, the STN dimensions between our anatomic and imaging studies presented only slight differences. Other minor weaknesses contain the inequality of right and left hemispheres numbers, as well as age and sex limitations.

## 5. Conclusion

Our study offers an insight into the anatomy and morphometry of the human STN resulted from detailed analysis of the measured data. Through two clinically oriented stereotactic studies, an imaging (MRI) and a gross anatomic, with totally 75 STNs studied, we measured STN dimensions at three different neurosurgically important three-dimensional levels. We provided evidence that the STN suffers age-related shrinkage. We found that the identification of the STN limits is easier on gross anatomic specimens than on MRIs and easier on T2-weighted MRIs compared to T1-weighted sections. We observed no significant difference of the STN dimensions between the two sexes and we confirmed that these values appear slightly smaller on MRIs than in anatomic specimens. Furthermore, we provided a reliable estimation of the AC-PC length. We hope that our work will be really useful to neuroscientists interested in STN anatomy.

## Figures and Tables

**Table 1:** Dimensions of the STN on T2-weighted transverse MRIs at level *Z* = −4, number of transverse slices where this nucleus was visible, *D* of the STN, and AC-PC length.

STN		*Z* = −4		Slices where STN was visible	*D* (mm)	AC-PC length (mm)	Age (years)	Gender
hemisphere		*W* (mm)	*L* (mm)					
1	R1	7.2	7.2	3	9.0	23.0	64	M
2	R2	4.5	6.3	3	8.1	21.6	70	M
3	R3	4.0	6.4	3	5.6	17.6	60	M
4	R4	5.0	7.5	3	9.0	27.0	69	F
5	R5	7.7	9.9	3	11.0	26.4	54	F
6	R6	5.0	6.0	4	8.0	26.0	71	F
7	R7	7.0	8.1	3	9.3	27.3	67	M
8	R8	5.0	8.0	3	8.0	27.0	69	M
9	R9	4.8	7.4	3	7.7	28.3	37	M
10	R10	8.9	11.2	3	10.3	27.9	51	F
11	R11	6.0	4.9	2	6.0	28.4	30	M
12	R12	8.4	10.5	4	12.1	24.7	51	M
13	R13	7.2	7.6	3	8.5	27.3	38	F
14	R14	—	—	—	—	27.9	55	F
15	R15	6.9	9.4	3	9.7	28.5	54	M
16	R16	—	—	3	8.8	27.2	23	M
17	R17	5.2	7.7	3	8.1	27.0	52	F
18	R18	8.7	6.7	3	9.8	29.3	67	M
19	R19	—	—	3	—	25.0	57	F
20	R20	4.0	9.6	4	9.3	25.4	63	M
21	R21	6.6	8.0	3	9.5	25.4	55	M
22	R22	4.8	6.8	3	6.5	25.6	71	F
23	R23	—	8.0	3	7.2	25.6	59	M
24	R24	4.9	7.8	4	7.9	28.2	36	M
25	R25	5.8	6.1	4	7.0	31.0	63	M
26	R26	6.2	8.5	3	8.9	25.4	38	F
27	L1	4.5	5.4	3	7.2	—	64	M
28	L2	4.5	6.8	3	8.1	—	70	M
29	L3	4.0	5.6	3	4.8	—	60	M
30	L4	5.6	6.9	3	9.0	—	69	F
31	L5	7.7	7.7	3	11.0	—	54	F
32	L6	4.0	6.0	4	8.5	—	71	F
33	L7	5.2	9.3	3	11.6	—	67	M
34	L8	6.0	6.0	3	—	—	69	M
35	L9	6.9	9.3	3	7.9	—	37	M
36	L10	8.8	9.1	3	9.6	—	51	F
37	L11	6.4	4.8	2	6.4	—	30	M
38	L12	7.3	9.4	4	11.1	—	51	M
39	L13	7.3	8.9	3	7.5	—	38	F
40	L14	—	—	—	—	—	55	F
41	L15	7.5	9.2	3	9.8	—	54	M
42	L16	—	—	3	9.2	—	23	M
43	L17	4.1	7.7	3	8.2	—	52	F
44	L18	7.8	9.3	3	9.3	—	67	M
45	L19	—	—	3	—	—	57	F
46	L20	7.2	11.0	4	9.8	—	63	M
47	L21	6.2	8.7	3	9.6	—	55	M
48	L22	4.7	7.0	3	6.7	—	71	F
49	L23	5.1	7.6	3	7.6	—	59	M
50	L24	4.2	7.5	4	7.9	—	36	M
51	L25	5.3	7.4	4	7.5	—	63	M
52	L26	5.0	5.6	3	6.9	—	38	F
**MV**		**5.98**	**7.73**	**3.16**	**8.52**	**26.31**	**54.77**	
**SD**		**1.46**	**1.56**	**0.47**	**1.56**	**2.67**	**13.60**	

STN: subthalamic nucleus; *W*: width; *L*: length; *D*: STN maximum dimension (diameter); AC: anterior commissure; PC: posterior commissure; R: right; L: left; M: male; F: female; MV: mean value; SD: standard deviation.

**Table 2:** Dimensions of the STN on transverse cerebral sections at level *Z* = −4 and AC-PC length.

STN		*Z* = −4			AC-PC length (mm)
hemisphere		*W* (mm)	*L* (mm)	*D* (mm)	
1	R3	6.0	5.0	6.0	23
2	R7	9.0	8.0	11.0	31
3	L8	6.0	7.0	8.0	31
4	L11	6.0	7.0	8.0	32
5	L9	7.0	6.0	9.0	27
6	L12	4.0	7.0	7.0	29
7	L10	6.0	8.0	8.0	29
8	L2	6.0	9.0	10.5	25
9	R2	6.0	8.0	9.0	26
10	L3	6.0	9.0	10.0	26
11	R5	6.0	7.0	7.0	26
12	R1	7.0	10.0	11.0	32
13	L6	7.0	10.0	10.0	30
14	R6	5.0	9.0	9.0	25
15	R4	7.0	9.0	10.0	25
16	L7	4.0	7.0	9.0	26
17	L1	7.0	6.0	9.0	24
18	L4	9.0	7.0	10.0	26
19	L5	6.0	10.0	10.0	26
20	L15	3.0	11.0	11.0	31
21	L16	10.0	9.0	11.0	28
22	L13	4.0	9.0	9.0	29
23	R9	6.0	7.0	9.0	26
24	L18	8.0	9.0	10.0	25
25	R16	—	—	—	26
26	L24	—	—	—	26
**MV**		**6.29**	**8.08**	**9.23**	**27.31**
**SD**		**1.65**	**1.50**	**1.37**	**2.59**

STN: subthalamic nucleus; *W*: width; *L*: length; *D*: maximum dimension (diameter) of the STN; AC: anterior commissure; PC: posterior commissure; R: right; L: left; MV: mean value; and SD: standard deviation.

## Abbreviations

AC: Anterior commissure
*D*: Maximum transverse dimension (diameter)
DBS: Deep brain stimulation
*H*: Height
*L*: Length
MCP: Mid-commissural point
MRI: Magnetic resonance image
MV: Mean value
PC: Posterior commissure
PD: Parkinson's disease
SD: Standard deviation
STN: Subthalamic nucleus
*W*: Width
3D: Three-dimensional.
